# Supplementary material for: Combined short-term and long-term emission controls improve air quality sustainably in China
Source: Nat Commun. 2024 Jun 17;15:5169. doi: 10.1038/s41467-024-49539-9 (PMC11183230; doi:10.1038/s41467-024-49539-9)
Supplement: Supplementary file 1 — Supplementary Information [file 41467_2024_49539_MOESM1_ESM.pdf]

Combined short-term and long-term emission controls improve air quality sustainably in China

Zhang Wen[1,2#], Xin Ma[1,#], Wen Xu[1,#], Ruotong Si[1], Lei Liu[1,3], Mingrui Ma[4], Yuanhong Zhao[5], Aohan Tang[1], Yangyang Zhang[1], Kai Wang[1], Ying Zhang[1], Jianlin Shen[6], Lin Zhang[7], Yu Zhao[4], Fusuo Zhang[1], Keith Goulding[8], Xuejun Liu[1, *]

[1]State Key Laboratory of Nutrient Use and Management, College of Resources and Environmental Sciences, National Academy of Agriculture Green Development, China Agricultural University, Beijing 100193, China

[2]State Environmental Protection Key Laboratory of Environmental Pollution and Greenhouse Gases Co-control, Chinese Academy of Environmental Planning, Beijing 100041, China

[3]College of Earth and Environmental Sciences, Lanzhou University, Lanzhou 730000, China

[4]State Key Laboratory of Pollution Control & Resource Reuse and School of Environment, Nanjing University, Nanjing 210008, China

[5]College of Oceanic and Atmospheric Sciences, Ocean University of China, Qingdao 266100, China

[6]Instute of Subtropical Agriculture, Chinese Academy of Sciences, Changsha 410125, China

[7]Laboratory for Climate and Ocean-Atmosphere Studies, Department of Atmospheric and Oceanic Sciences, School of Physics, Peking University, Beijing 100871, China

[8]Sustainable Soils and Crops, Rothamsted Research, Harpenden AL5 2JQ, UK

[#] These authors contribute to this work equally.

Correspondence to: Xuejun Liu (liu310@cau.edu.cn)

Note S1 Difference-in-Differences (DID) method

We used the DID method to quantitatively examine whether and to what extent the short-term emission control measures influenced long-term policies. DID recognizes the NC1(Beijing) as the treatment group, while the other sites are regarded as the control group. Differences for each group in $PM_{2.5}$ concentrations, oxidized N deposition, reduced N deposition during APEC, and Parade (emissions data of Olympics events were lacking, and the limited data available after the implementation of short-term measures during the BRS period was therefore not used in the assessment) when compared before and after the implementation of short-term measures are then calculated. According to the Random Forest assessment (in Methods), the important variables were found to be emission data (SO_2 emission, NO_X emission, NH_3 emission), meteorological parameters (2m temperature, atmospheric pressure, 10m wind speed, boundary layer height) and NDVI. The changes in these differences thus represent the net effect on $PM_{2.5}$ concentration, oxidized N deposition, and reduced N deposition brought by the short-term measures, and statistically significant differences were set at p values < 0.05. The DID method requires there to be a similarity between the treatment group and the control group. In response to this, parallel trends were tested by the event analysis method and p values> 0.05 indicated that there was similarity. All actions were performed in Stata MP version 18.

Note S2 The assessment of "NH_3-rich" or "NH_3-poor"

The "NH_3-rich atmospheric environment" here represents a relatively high concentration of NH_3 in the atmosphere. We added the categorization of "NH_3-rich" or "NH_3-poor" to further quantify the degree of "NH_3-richness"[1]. The equations as follows:

$$AdjGR = \frac{free\ ammonia}{total\ nitrate} = \frac{TA - DSN \times TS}{TN} = \frac{[NH_3](g) + [NO_3^-](p)}{[NO_3^-](p) + [HNO_3](g)} \quad (1)$$

$$DSN = \frac{[NH_4^+](p) - [NO_3^-](p)}{[SO_4^{2-}](p)} \quad (2)$$

$$TA = [NH_3](g) + [NH_4^+](p) \quad (3)$$

$$TN = [HNO_3](g) + [NO_3^-](p) \quad (4)$$

$$TS = [SO_2](g) + [SO_4^{2-}](p) \quad (5)$$

The concentration unit of gaseous and particulate pollutants is μmol m^{-3}. Where an $AdjGR$ of <1 indicates an NH_3-poor regime, where small perturbations in NH_3 emissions would have a significant effect on SIAs. Likewise, an $AdjGR$ of >1 indicates NH_3-rich conditions with a free NH_3 ratio capable of neutralizing excess HNO_3 produced by the additional increase in NOx emission.

The national average value of $AdjGR$ is 3.24, which is more than 1, so we consider that our country generally has an NH_3-rich atmospheric environment.

Note S3 Sample collection and processing

Samplers for collecting Nr gases and particulates were placed 2 m above the ground. The adsorbents for NH_3 and pNH_4^+ were citric acid or phosphoric acid; those for HNO_3 and pNO_3 were potassium hydroxide and for NO_2 we used triethanolamine. Precipitation samples were collected in a funnel-shaped glass bucket, and a graduated cylinder was used to measure the precipitation amount. After each precipitation event (8:00 am-8:00 am the next day), the collected samples were transferred into a 50 ml clean polyethylene bottle and the rain gauge was cleaned with high-purity water to avoid cross-contamination.

Gaseous and particulate Nr samples were collected monthly. After sampling, all exposed samplers and samples were returned to the laboratory at China Agricultural University for chemical analysis. DELTA samplers and passive samples were stored at 4ºC before analysis. HNO_3 denuders and alkaline-coated filters were extracted with 10 mL 0.05% H_2O_2. High-purity water was used to extract the NH_3 denuders, acid-coated filters, and ALPHA samplers. NH_4^+ and NO_3^- in the extracts were measured using an AA3 continuous-flow analyzer (Bran + Luebbe GmbH, Norderstedt, Germany). The Gradko samplers were extracted with an aqueous solution containing sulfanilamide, phosphoric acid, and N-1-Naphthylethylene-diamine and analyzed for NO_2^- colorimetrically at a wavelength of 542 nm. Laboratory and field blanks were co-processed to calibrate the samples in every experiment.

Each collected precipitation sample was filtered using a 0.45 μm syringe and 10 ml of filtrate was retained. NH_4^+-N and NO_3^--N in the filtrate were determined on the AA3

continuous-flow analyzer described above.

Note S4 Simulation of dry deposition velocities of Nr species

The dry deposition of gaseous and particulate Nr species was calculated by multiplying measured concentrations with simulated deposition velocities (*Vd*) from the GEOS (Goddard Earth Observing System)-Chem chemical transport model (http://geos-chem.org). The model is driven by GEOS (Goddard Earth Observing System) assimilated meteorological data from the NASA Global Modeling and Assimilation Office (GMAO). GEOS-5 data are available with a temporal resolution of 6 h (3 h for surface variables and mixing depths) and a horizontal resolution of 1/2° latitude × 2/3° longitude. The nested-grid version of GEOS-Chem was used with the native 1/2° × 2/3° resolution over East Asia (70°E–150°E, 11°S–55°N). Calculation of dry deposition of Nr species follows a standard big-leaf resistance-in-series model for gases and aerosols. The aerodynamic resistance to turbulent transfer from the measurement heights (~2 m) to the roughness height is estimated using the GEOS-5 data. The surface uptake resistance is calculated based on the Global Land Cover Characteristics Database Version 2.0 (http://edc2.usgs.gov/glcc/globdoc2_0.php), which defines land types (e.g., urban and forest) at 1 km × 1 km resolution and is then binned to the model resolution as a fraction of the grid cell covered by each land type. The model 1/2° resolution thus coarsely represents regional land characteristics around the monitoring sites. Bi-directional NH_3 exchange is not considered in the model.

Table S1 Control measures implemented in Beijing-Tianjin-Hebei during the Olympic Games, APEC, Military Parade, and BRS[2]

Events	Provinces	Control measures	
Olympic Games (Aug. 8–24, 2008)	Beijing	1	Strict and provisional driving restrictions on motor vehicles by odd-even number (for NO_X and $PM_{2.5}$);
		2	Suspended the use of 70% of government motor vehicles (for NO_X and $PM_{2.5}$);
		3	Stopped some construction site operations (for SO_2 and $PM_{2.5}$);
		4	More effective road cleaning (for $PM_{2.5}$);
		5	Stopped production and limited production in key polluting enterprises (for SO_2 and NO_X);
		6	Emission pollutants (SO_2, NO_X, etc.) decreased by 30% from fuel combustion (for SO_2 and NO_X);
		7	Organic emissions at gas stations, tankers, and storage depots reduced (for $PM_{2.5}$);
	Tianjin	1	Strict and provisional driving restrictions on motor vehicles by odd-even number (for NO_X and $PM_{2.5}$);
		2	Some gas stations in key areas stopped refueling operations during key hours (for NO_X and $PM_{2.5}$);
		3	Open burning of straw strictly prohibited (for $PM_{2.5}$ and NH_3);
		4	Non-electricity coal-fired facilities and small thermal power plants ceased operation (for SO_2 and NO_X);
		5	Selected pollutant emissions suspended (for SO_2 and NO_X);
		6	Increased road flushing and sprinkling for dust suppression (for $PM_{2.5}$);
	Hebei	1	All production activities related to construction works are suspended (for $PM_{2.5}$);
		2	Increase in the frequency of road water spraying (for $PM_{2.5}$);
		3	Strict control of straw burning and coal burning (for $PM_{2.5}$, NO_X, SO_2, and NH_3);
		4	Strengthened the inspection of vehicles entering Beijing (for NO_X and $PM_{2.5}$).

APEC (Nov. 1–12, 2014)	Beijing	1	Strict supervision and driving restrictions on motor vehicles (for NO_X and $PM_{2.5}$);
		2	Driving ban on 70% of vehicles belonging to government and state-owned enterprises (for NO_X and $PM_{2.5}$);
		3	All construction sites within the fifth ring road suspended (for $PM_{2.5}$);
		4	Production by coal-fired and industrial companies suspended (for SO_2 and NO_X);
		5	Emission standard of pollutants (SO_2, NO_X, etc.) increased by 50% (for SO_2 and NO_X);
		6	Key roads required to be cleaned at a high frequency every day (for $PM_{2.5}$);
		7	Strict control of express deliveries in Beijing (for $PM_{2.5}$);
	Tianjin	1	50% of motor vehicles limited and public transit capacity enhanced (for NO_X and $PM_{2.5}$);
		2	All production activities related to construction works suspended (for $PM_{2.5}$);
		3	Emission standard of pollutants (SO_2, NO_X, etc.) increased by 30% (for SO_2 and NO_X);
		4	Main roads in the central area cleaned once a day (for $PM_{2.5}$);
		5	Strict control of straw burning and coal burning (for $PM_{2.5}$ and NH_3);
	Hebei	1	Strengthened inspection of vehicles entering Beijing (for NO_X and $PM_{2.5}$);
		2	Real-time monitoring of straw burning and (for $PM_{2.5}$ and NH_3);
		3	Emission limits for pollutants increased by 50% in Shijiazhuang (for SO_2, NO_X and $PM_{2.5}$);
		4	Construction sites and key enterprises suspended or restricted in key areas (for SO_2, NO_X and $PM_{2.5}$).
Military Parade (Sep. 3, 2015)	Beijing	1	Strict and provisional driving restrictions on motor vehicles by odd-even number (for NO_X and $PM_{2.5}$);
		2	Driving ban on 80% of vehicles belonging to government and state-owned enterprises (for NO_X and $PM_{2.5}$);

		3	Widespread surprise inspections and rectification of polluting enterprises (for SO_2, NO_X and $PM_{2.5}$);
		4	All construction works within the fifth ring road suspended (for $PM_{2.5}$);
		5	Removed from use 217,000 old vehicles and 244 heavy polluting enterprises in advance (for SO_2, NO_X and $PM_{2.5}$);
		6	58 important roads cleaned at high frequency every day (for $PM_{2.5}$);
		7	Enhanced supervision of thermal power plants and steel companies in west and south Beijing based on scientific research (for SO_2, NO_X and $PM_{2.5}$);
	Tianjin	1	Increased emission reduction standard of coal power plants by more than 50% (for SO_2);
		2	Completed the yellow label car elimination task six months in advance (for NO_X and $PM_{2.5}$);
		3	Strengthened efforts to rectify environmental pollution incidents (for SO_2, NO_X and $PM_{2.5}$);
		4	Cars that did not meet national emission standards were not allowed to enter Beijing (for NO_X and $PM_{2.5}$);
		5	Main roads in the central area and Binhai area required to be cleaned once a day (for $PM_{2.5}$);
	Hebei	1	Set up an inspection team to account for environmental violations (for SO_2, NO_X and $PM_{2.5}$);
		2	Driving restrictions on motor vehicles by odd-even number (for NO_X and $PM_{2.5}$);
		3	Production of key companies suspended in Baoding and other cities (for SO_2, NO_X and $PM_{2.5}$);
		4	Enhanced the desulfurization facilities in ANSTEEL in Handan (for SO_2, NO_X and $PM_{2.5}$);
		5	All construction works and pollutant companies suspended or restricted (for SO_2, NO_X and $PM_{2.5}$).
Belt and Road (May. 8–17, 2017)	Beijing	1	The principle of "non-disturbance of the resident";
		2	Provisional traffic restrictions on vehicles with dangerous chemicals (for NO_X and $PM_{2.5}$);
		3	Some logistics vehicles are completely banned (for NO_X and $PM_{2.5}$);

	4	Enterprises that could not meet emissions standards or with excessive emissions suspended (for SO_2, NO_X and $PM_{2.5}$);
	5	Set up a special inspection team to ensure air quality (for SO_2, NO_X and $PM_{2.5}$);
Tianjin	1	Strengthened control of coal pollution, industrial pollution and dust pollution (for SO_2, NO_X and $PM_{2.5}$);
	2	Strengthened the assessment and accountability mechanism;
Hebei	1	10-day corporate inspection (for SO_2, NO_X and $PM_{2.5}$);
	2	Strengthened environmental law enforcement and data monitoring (for SO_2, NO_X and $PM_{2.5}$).

Table S2 Scenarios of sensitivity of $PM_{2.5}$ and N deposition to emission changes[3]

	**Meteorological conditions**	**Emission scenarios**	**Introduction**
Base	FNL_2017	MEIC 2017	Base
Case1	RCP4.5_2030	DPEC 2030 NG	Carbon-neutral climate goals combined with enhanced pollution control policies
Case2	RCP4.5_2060	DPEC 2060 NG	

Table S3 Duration of pre-event, event window and post-event for each event

	**Pre-event**	**Event window**	**Post-event**
**Event 1**: Olympics Games (Olympic)	Jun. 1-30, 2008	Aug. 8-24, 2008	Sep. 24-Oct. 23, 2008
**Event 2**: Asia-Pacific Economic Cooperation Summit (APEC)	Oct. 1-31, 2014	Nov. 1-12, 2014	Nov. 13-Dec. 31, 2014
**Event 3**: Military Parades (Parade)	Aug. 3-19, 2015	Aug. 20-Sep. 3, 2015	Sep. 3-29, 2015
**Event 4**: Belt and Road Summit (BRS)	Apr. 28-May 7, 2017	May 8-17, 2017	May 18-Jun. 17, 2017

Table S4 Description of China's Nationwide Nitrogen Deposition Monitoring Network (NNDMN) sites

**Sub-region**	**Sites**	**Longitude (E°)**	**Latitude (N°)**	**Land-use type**	**Sampling period**	
					**Dry deposition**	**Bulk deposition**
North China	NC1	116.28	40.02	Urban	2011-2020	2011-2020
	NC2	116.37	39.96	Urban	2012-2018	2012-2020
	NC3	113.63	34.75	Urban	2011-2020	2011, 2015-2020
	NC4	120.40	36.32	Urban	2011, 2015-2020	2011-2012, 2015-2020
	NC5	115.48	38.85	Rural	2011-2020	2011-2020
	NC6	116.20	40.11	Rural	2011-2020	2011-2020
	NC7	116.10	39.72	Rural	2011-2020	2011-2013, 2016
	NC8	116.00	40.50	Rural	2015-2020	2015-2020
	NC9	114.94	36.78	Rural	2011-2020	2011-2020
	NC10	112.89	38.05	Rural	2011-2020	2011-2020
	NC11	112.69	37.56	Rural	2016-2020	2016-2020
	NC12	108.01	34.31	Rural	2011-2019	2011-2014, 2017-2019
	NC13	107.15	34.38	Rural	2017-2020	2017-2020
	NC14	116.63	36.94	Rural	2014-2020	2014-2020
	NC15	114.07	31.83	Background	2014-2017	2014-2017
	NC16	120.75	37.93	Background	2011-2020	2011-2020
	NC17	120.18	35.77	Background	2011-2020	2011-2020
Northwest China	NW1	102.60	38.07	Rural	2011-2020	2011-2019
	NW2	116.49	42.20	Background	2013-2020	2013-2020
	NW3	83.71	42.88	Background	2011, 2015-2020	2011, 2019-2020
	NW4	87.56	44.17	Background	2015-2020	2017-2020
Northeast China	NE1	121.58	38.92	Urban	2011-2017	2011-2017
	NE2	124.17	43.36	Rural	2011-2020	2011-2020
	NE3	124.83	43.53	Rural	2011-2020	2011-2020

	NE4	127.59	44.77	Rural	2015-2020	/
Southeast China	SE1	118.97	32.12	Urban	2015-2020	2015-2020
	SE2	113.27	23.16	Urban	2011-2020	2011-2020
	SE3	121.53	29.61	Rural	2011-2020	2011-2020
	SE4	119.22	32.12	Rural	2011-2020	2011-2020
	SE5	119.20	31.81	Rural	2015-2020	2015-2020
	SE6	120.57	32.37	Rural	2016-2018	2016-2018
	SE7	117.56	32.88	Rural	2014-2020	2014-2020
	SE8	115.79	30.01	Rural	2012-2019	2012-2019
	SE9	112.38	30.72	Rural	2016-2019	2016-2019
	SE10	113.09	28.19	Rural	2015-2018	/
	SE11	111.97	28.61	Rural	2011-2020	2011-2020
	SE12	113.41	28.21	Rural	2016-2019	/
	SE13	113.41	28.52	Rural	2011-2020	2011-2020
	SE14	113.34	28.56	Rural	2011-2020	2011-2020
	SE15	110.01	27.56	Rural	2017-2020	2017-2020
	SE16	110.33	21.26	Rural	2011-2020	2011-2020
	SE17	119.36	26.17	Rural	2011-2020	2011-2020
	SE18	116.57	25.13	Rural	2016-2020	2011-2020
	SE19	117.19	16.90	Rural	2016-2020	2011-2020
	SE20	116.35	25.83	Rural	2016-2020	2011-2018
	SE11	113.31	28.61	Background	2011-2020	2011-2020
	SE22	117.98	27.63	Background	2017-2020	2017-2020
	SE23	118.84	32.07	Background	2017-2018	2017-2018
Southwest China	SW1	104.08	30.64	Urban	2015-2020	2015-2020
	SW2	103.84	30.55	Rural	2011-2020	2011-2020
	SW3	104.04	30.61	Rural	2015-2020	2015-2020
	SW4	105.39	31.22	Rural	2011-2017	2011-2017
	SW5	106.18	29.06	Rural	2013-2020	2013-2020
	SW6	107.90	23.05	Rural	2014-2018	2014-2018
	SW7	101.57	21.46	Rural	2017-2020	2017-2020
	SW8	102.13	29.65	Background	2015-2020	2015-2020

Qinghai-Tibet Plateau	TP1	101.79	36.62	Urban	2014-2020	2014-2020
	TP2	94.36	29.65	Rural	2011, 2015-2020	2011, 2015-2020
	TP3	94.49	29.57	Background	2016-2019	2016-2019

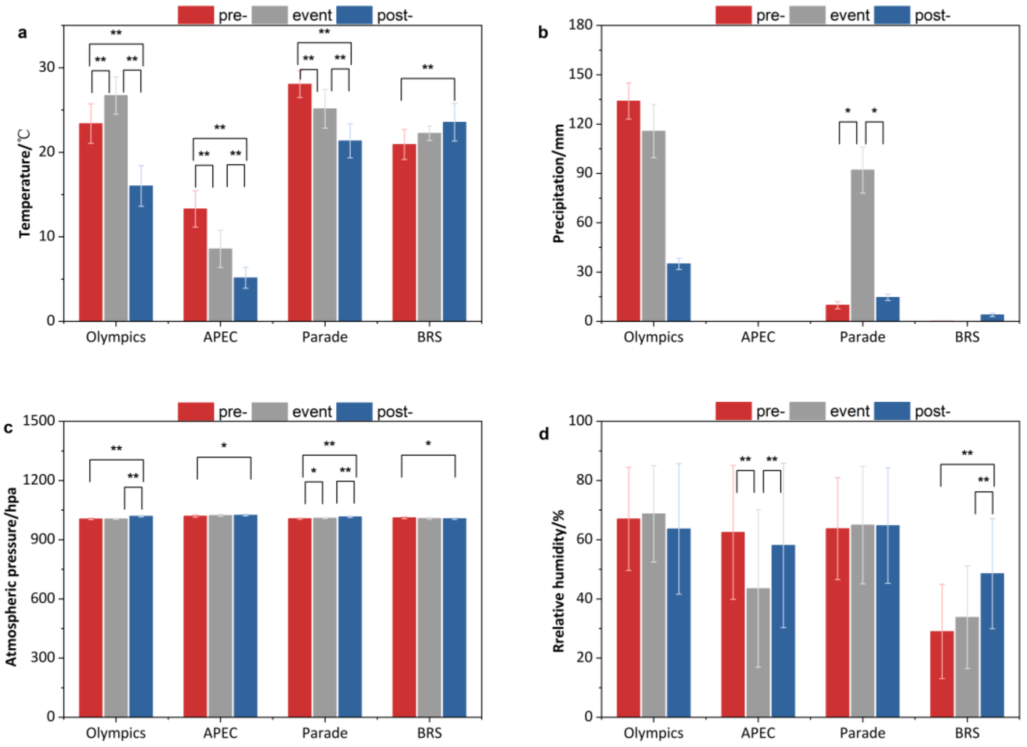

Figure S1 Differences in meteorological factors (average ± SD). **a**: Temperature, **b**: precipitation, **c**: atmospheric pressure, **d**: relative humidity during the Olympics, APEC, Parade, and BRS. *: p<0.05, **: p<0.01. Source data are provided as a Source Data file.

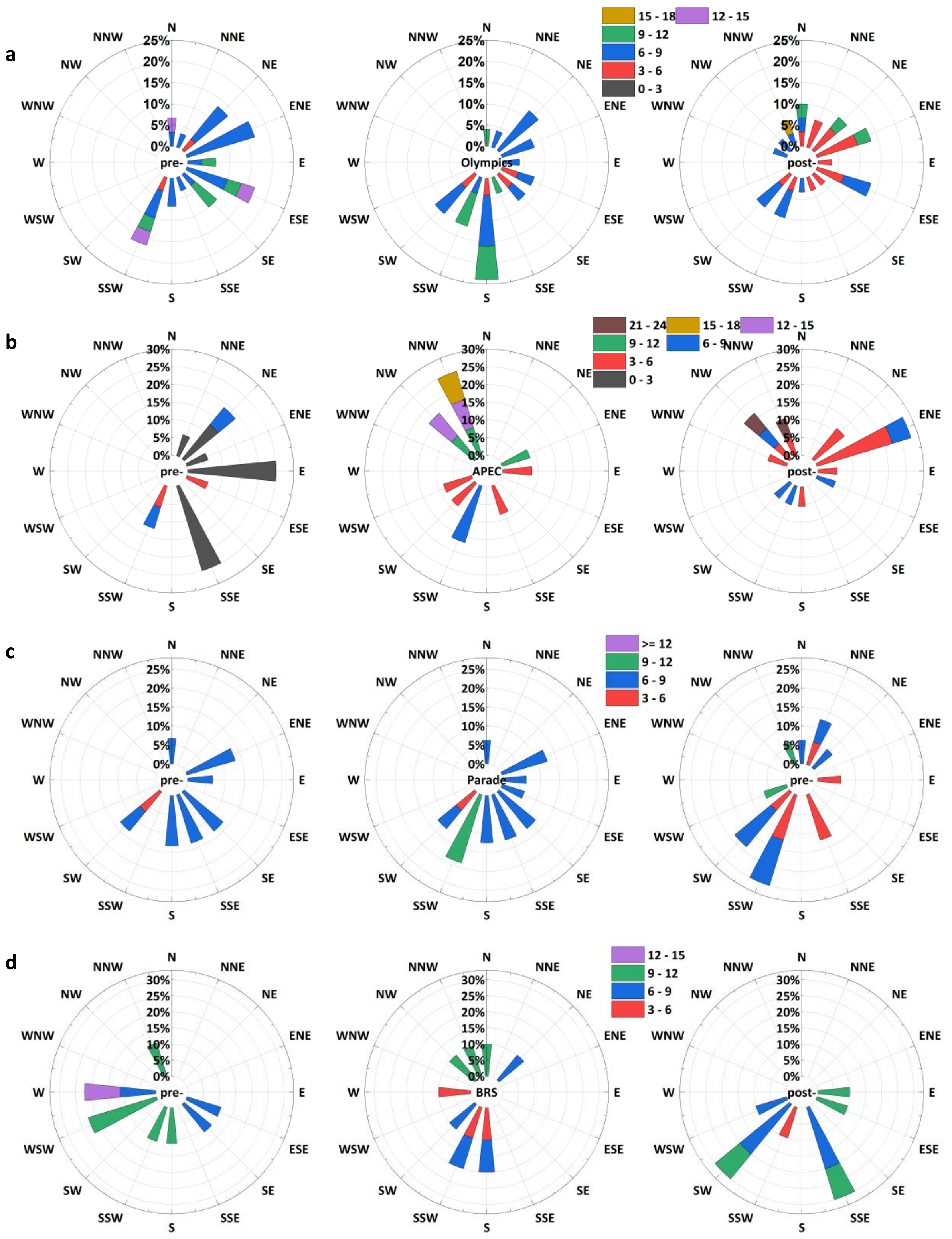

Figure S2 Differences in wind speed (km/h) and direction. **a**: Olympics, **b**: APEC, **c**: Parade, **d**: BRS, denoting three subperiods of the pre-event (left), event (middle), and post-event (right), respectively. Source data are provided as a Source Data file.

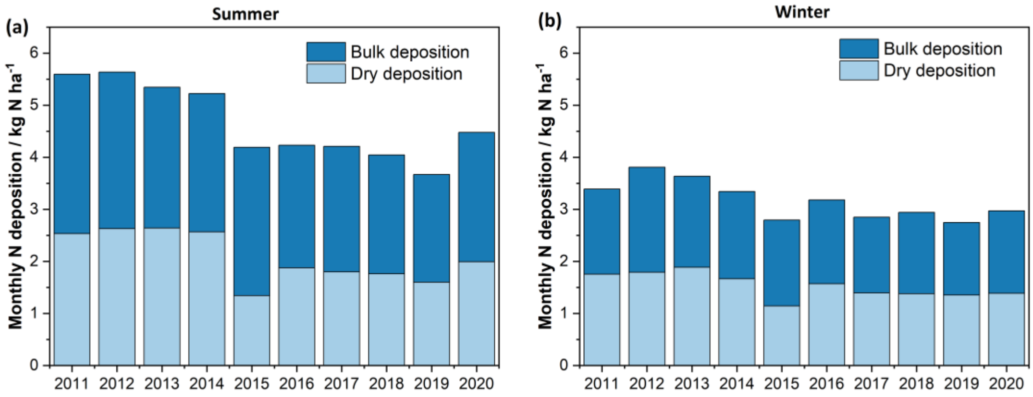

Figure S3 Average monthly values of atmospheric N deposition (including bulk deposition and dry deposition) in summer (**a**) and winter (**b**) from 2011 to 2020. Source data are provided as a Source Data file.

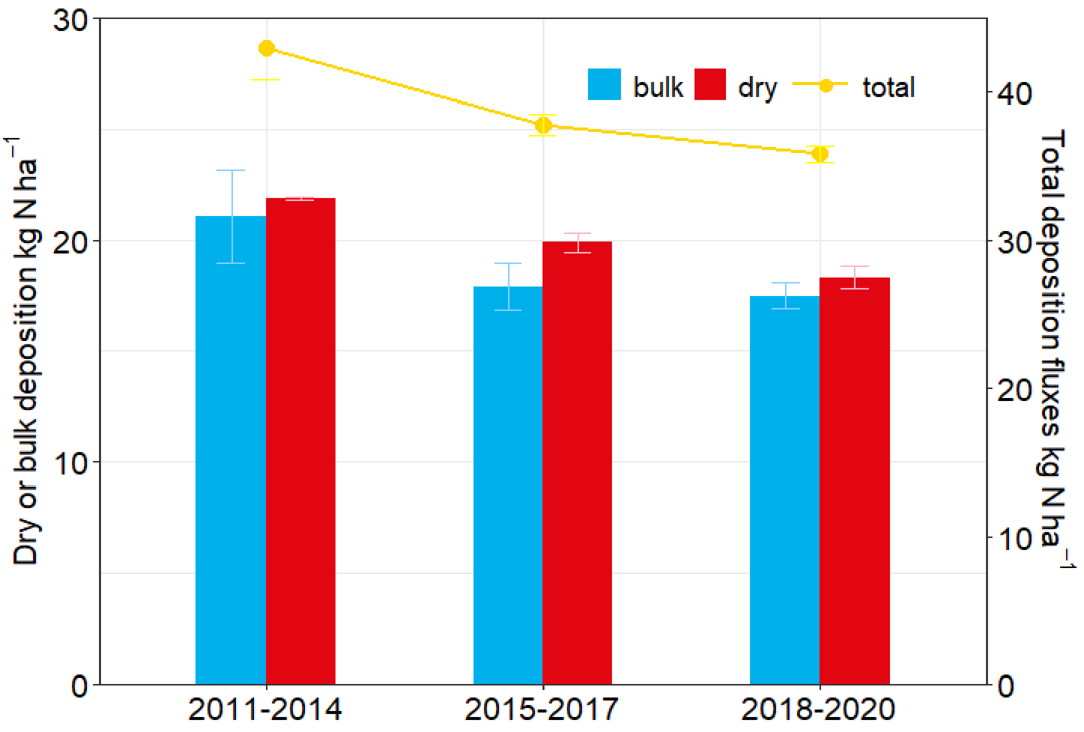

Figure S4 Comparison of N deposition (average ± SD) over three sampling dates (2011-2014, 2015-2017, 2018-2020). Source data are provided as a Source Data file.

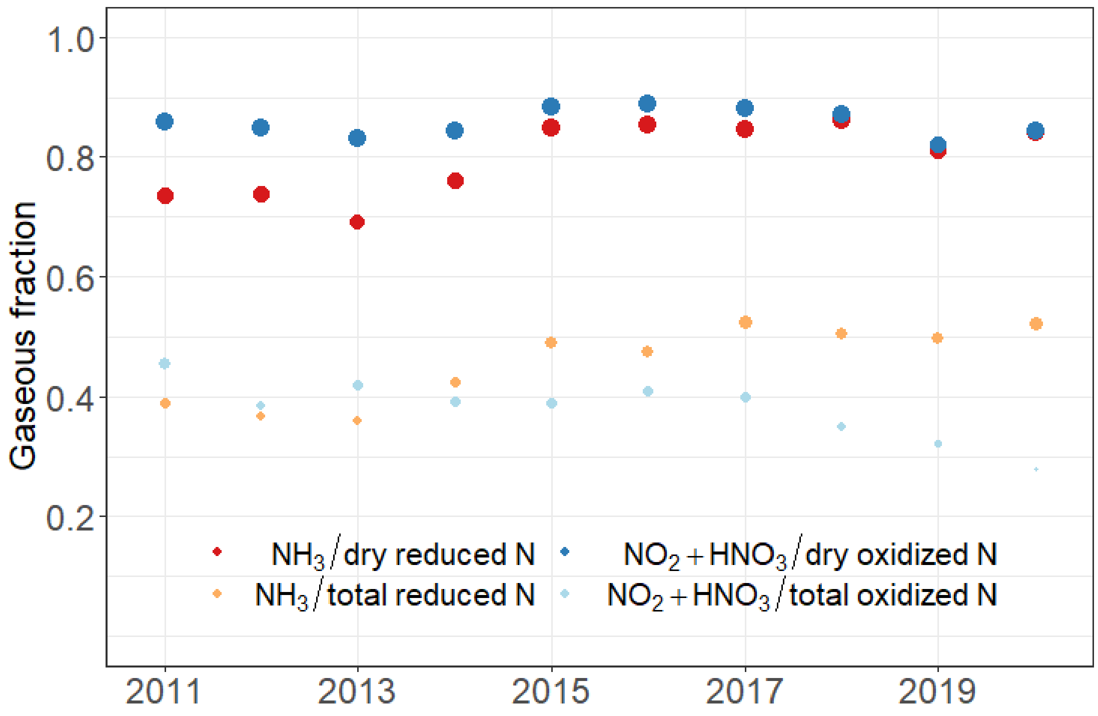

Figure S5 Gaseous fraction of NH_3 in dry reduced N deposition and total reduced N deposition; NO_2 and HNO_3 in oxidized N deposition and total oxidized N deposition. Source data are provided as a Source Data file.

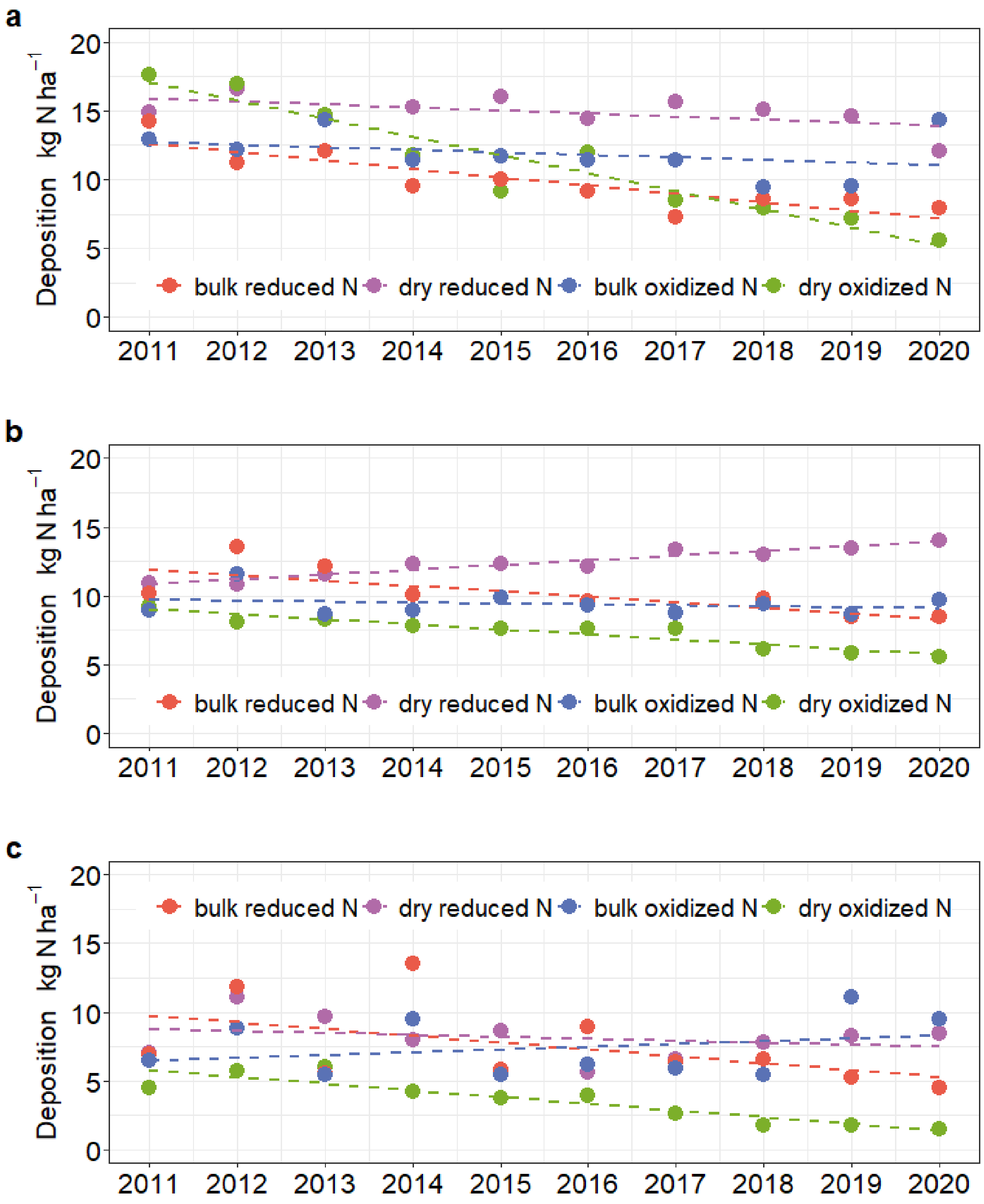

Figure S6 Interannual variation of nitrogen deposition for three land-use types. **a**: urban, **b**: rural, **c**: background). Source data are provided as a Source Data file.

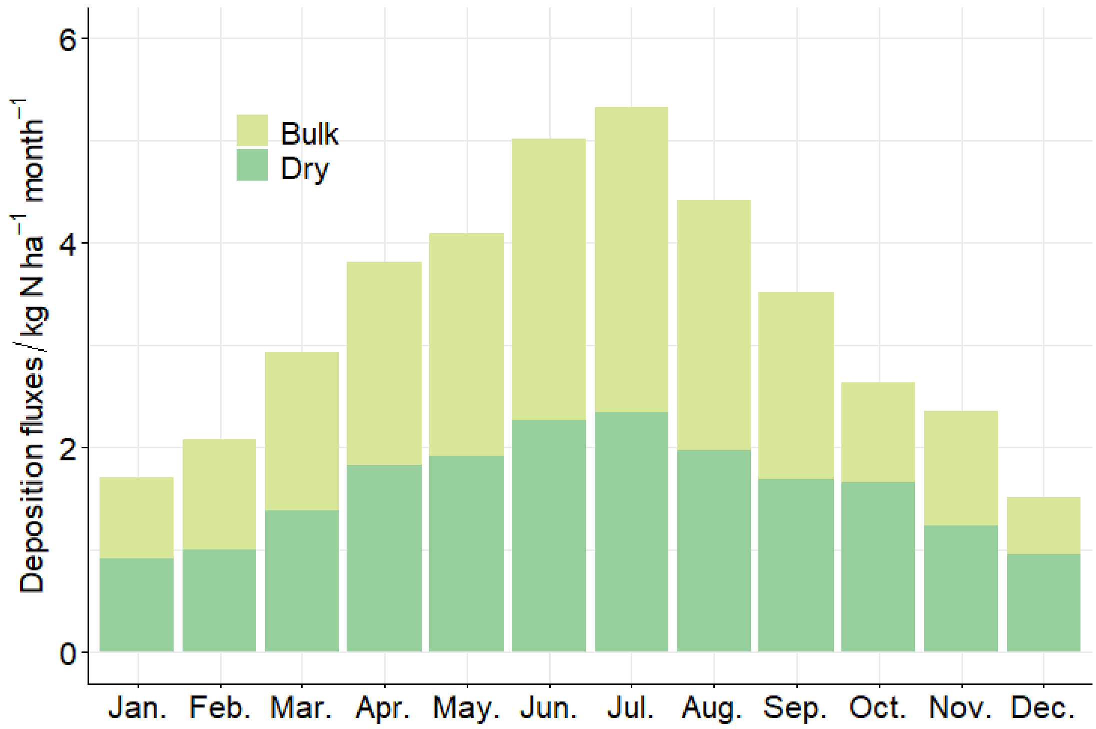

Figure S7 Monthly variation of dry and bulk nitrogen deposition. Source data are provided as a Source Data file.

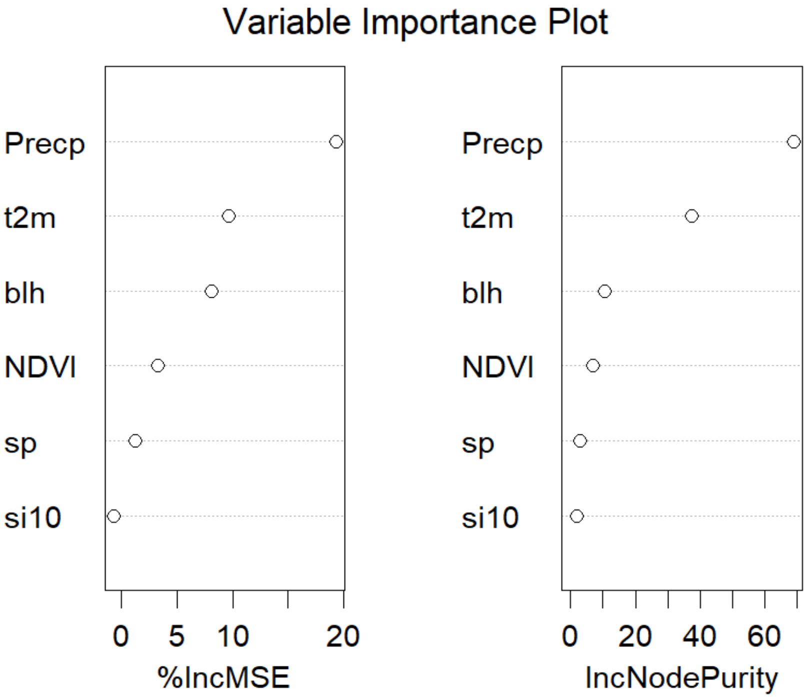

Figure S8 The effect of variables on nitrogen deposition.

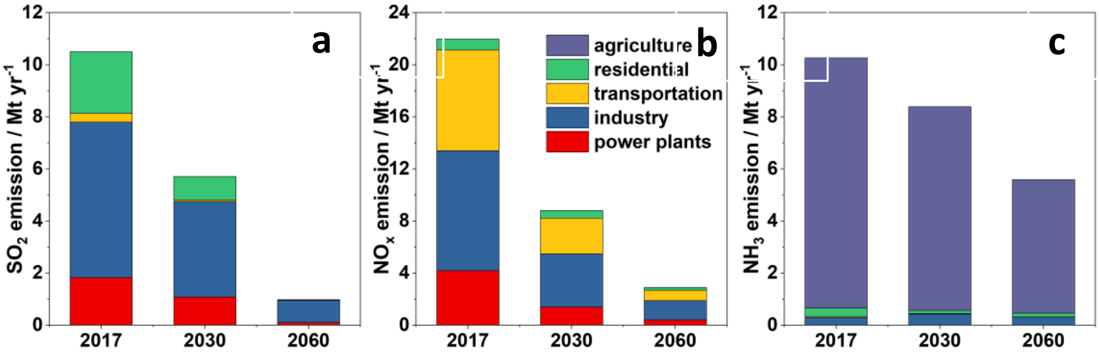

Figure S9 Changes in acid gas (**a**: NO_x and **b**: SO_2) and **c**: NH_3 emissions in 2030 and 2060 compared with 2017. Source data are provided as a Source Data file.

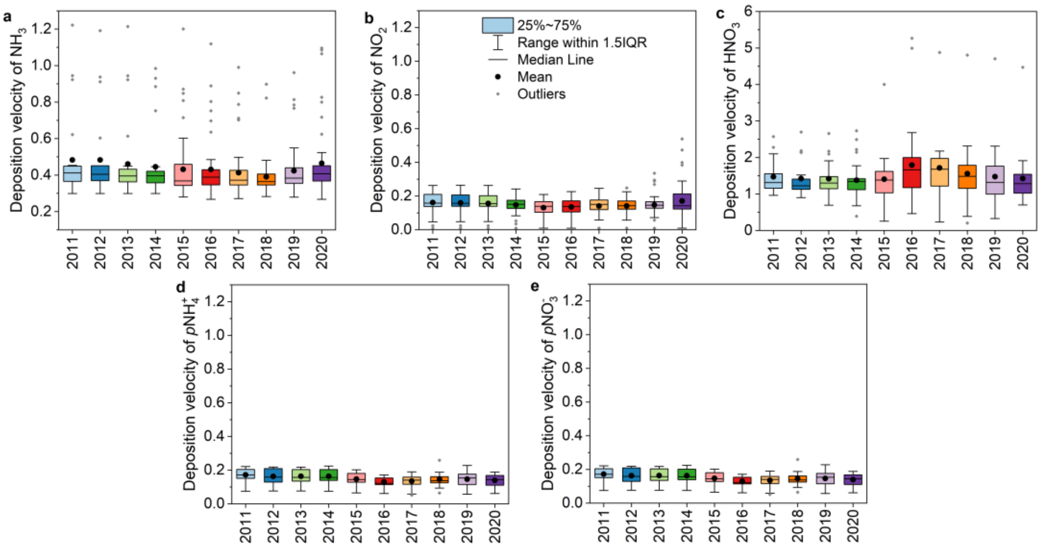

Figure S10 Variations in the dry deposition velocities of Nr species. **a**: NH_3, **b**: NO_2, **c**: HNO_3, **d**: pNH_4^+, **e**: pNO_3^-) at sites within the National Nitrogen Deposition Monitoring Network (NNDMN) from 2011 to 2020. Source data are provided as a Source Data file.

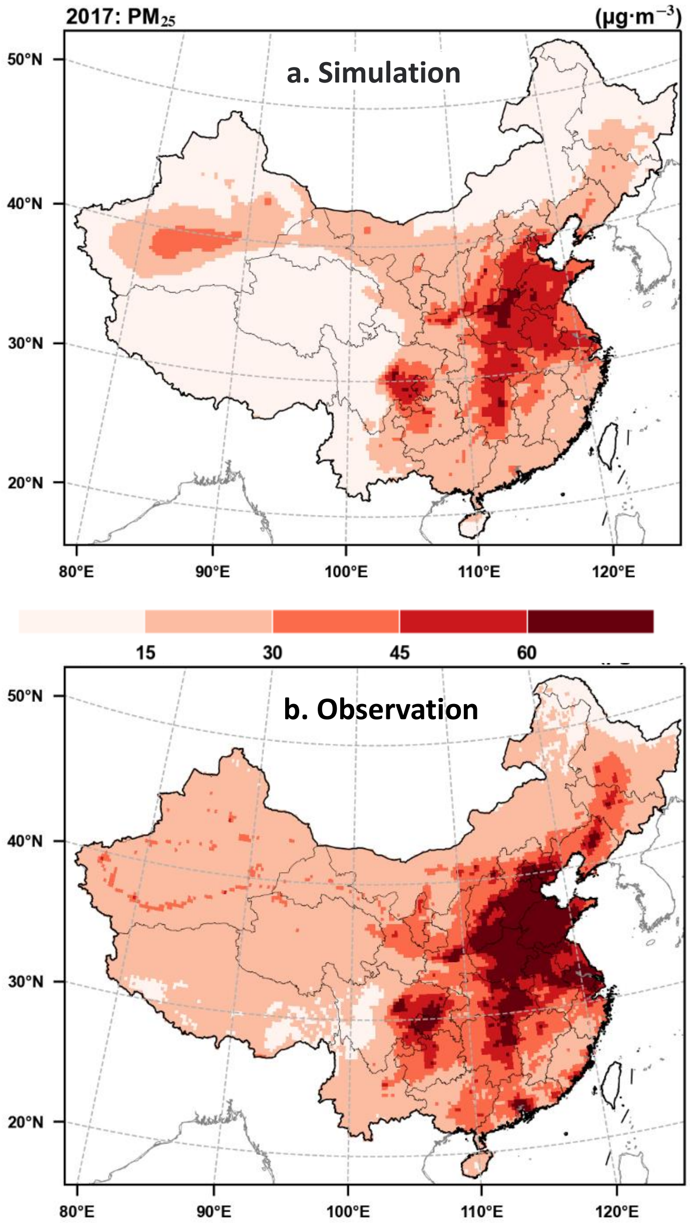

Figure S11 Comparison of simulated (**a**) and observed (**b**) $PM_{2.5}$ concentrations in China in 2017. The map data are provided by the Resource and Environment Science and Data Platform (Xinliang Xu. Multi-year provincial administrative division boundary data in China. http://www.resdc.cn/DOI, 2023. The DOI: 10.12078/2023010103).

Supplementary References

1 Pinder, R.W., Dennis, R.L., Bhave, P.V. Observable indicators of the sensitivity of $PM_{2.5}$ nitrate to emission reductions—Part I: Derivation of the adjusted gas ratio and applicability at regulatory-relevant time scales. *Atmos. Environ.* **42**, 1275-1286 (2008).

2 Ma, X., Li, C., Dong, X. & Liao, H. Empirical analysis on the effectiveness of air quality control measures during mega events: Evidence from Beijing, China. *J. Clean Prod.* **271**, 122536 (2020).

3 Tong, D. et al. Dynamic projection of anthropogenic emissions in China: methodology and 2015–2050 emission pathways under a range of socio-economic, climate policy, and pollution control scenarios. *Atmos. Chem. Phys.* **20**, 5729-5757 (2020).
